# Supplementary material for: Quality Assessment of Processed Graphene Chips for Biosensor Application
Source: Materials (Basel). 2023 Aug 15;16(16):5628. doi: 10.3390/ma16165628 (PMC10456491; doi:10.3390/ma16165628)
Supplement: Supplementary file 1 [file materials-16-05628-s001.zip › materials-2443628-supplementary.pdf]

Supplementary Materials
For

Quality assessment of processed graphene chips for biosensor application

Natalia M. Shmidt[1], Evgeniya I. Shabunina[1], Ekaterina V. Gushchina[1], Vasiliy N. Petrov[1], Ilya A. Eliseev[1], Sergey P. Lebedev[1], Sergei Iu. Priobrazhenskii[1] , Elena M. Tanklevskaya[1], Mikhail V. Puzyk[2], Alexander D. Roenkov[3], Alexander S. Usikov[3], Alexander A. Lebedev[1,*]

[1] Ioffe Institute, 26 Politekhnicheskaya, St Petersburg 194021, Russian Federation
[2] Herzen State Pedagogical University of Russia, 191186 St. Petersburg, Russia
[3] Nitride Crystals Group, 194156 St. Petersburg, Russia

* Correspondence: shura.lebe@mail.ioffe.ru (A.A.L.)

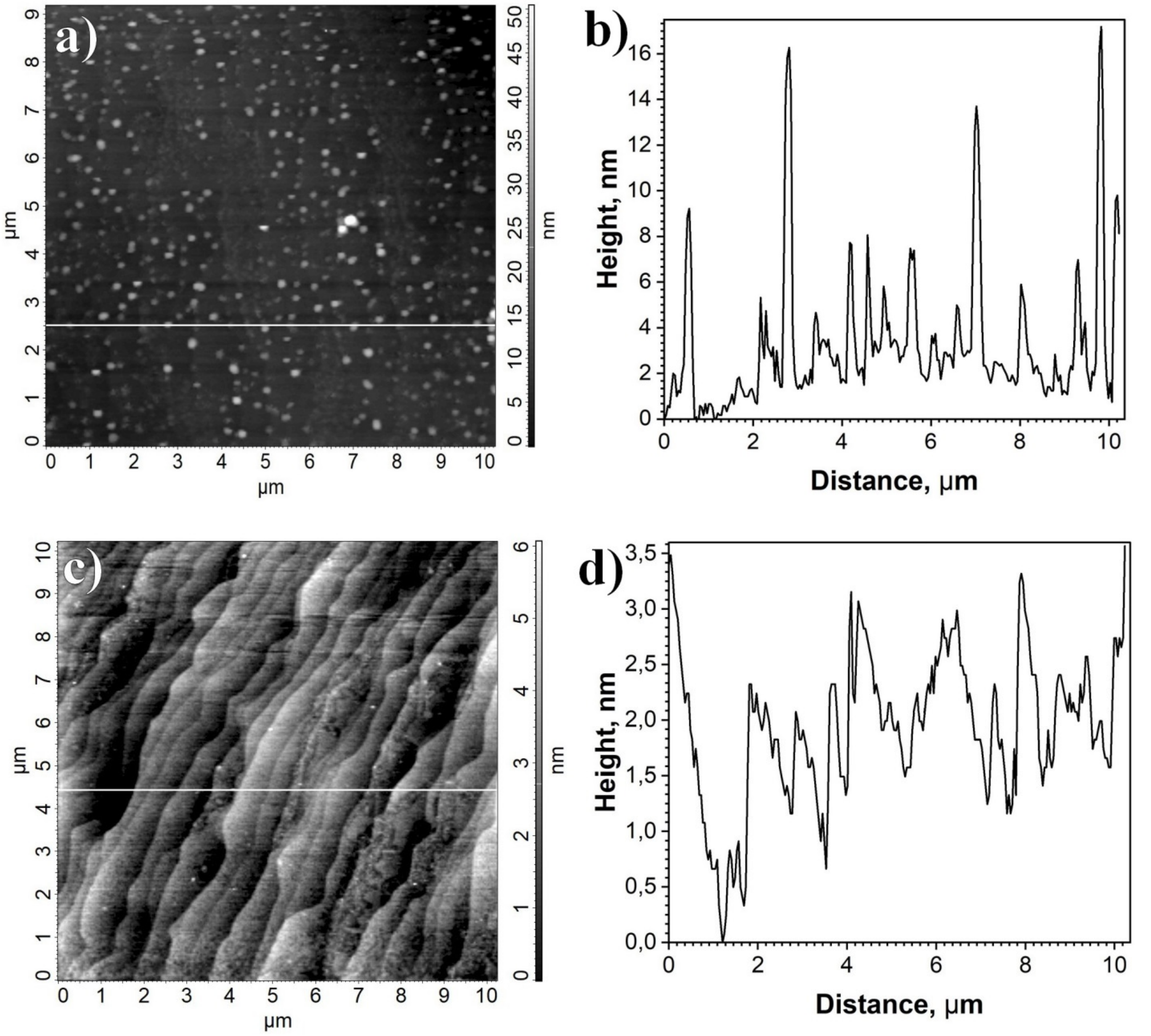

Figure S1. Graphene surface topography in chips by AFM method (scan: 10 μm × 10 μm).
(a) After PLG without cleaning LRRs (RMS = 3.40 nm); typical change in the surface topography of graphene caused by the formation of LRRs visible as white local regions in AFM images is observed. A thin layer of photoresist between terraces prevents the observation of terraces of the processed graphene on the AFM image.
(b) The graphene surface profile along the white line in a topographic image (a). The profile height reaches more than 20 nm.
(c) After PLG with additionally cleaned LRRs (RMS = 0.52 nm); The terraces on the graphene surface are visible
(d) The graphene surface profile along the white line in a topographic image (c). The profile height is less than 4 nm.

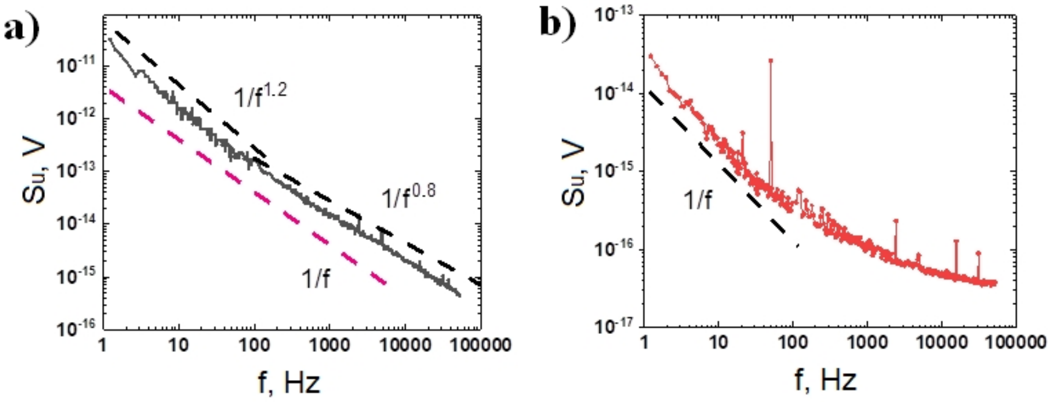

Figure S2. Frequency dependence of the S_U for processed chips with different S_U values. Dushed lines are simulation of the 1/f dependences.

(a) after PLG without cleaning LRRs (RMS = 3.40 nm) with the region of dependence as S_U `~ $1/f^{1.2}$; $S_U(f)$ dependences of this type indicate the presence of inhomogeneously deformed regions of graphene

(b) after PLG with additionally cleaned LRRs (AFM RMS = 0.52 nm); A weaker dependence S_U ~$1/f^{0.8}$ is observed for higher frequencies f>100 Hz. We believe the S_U dependence at a higher frequency may be due to a superposition of 1/f noise and generation-recombination (GR) noise.

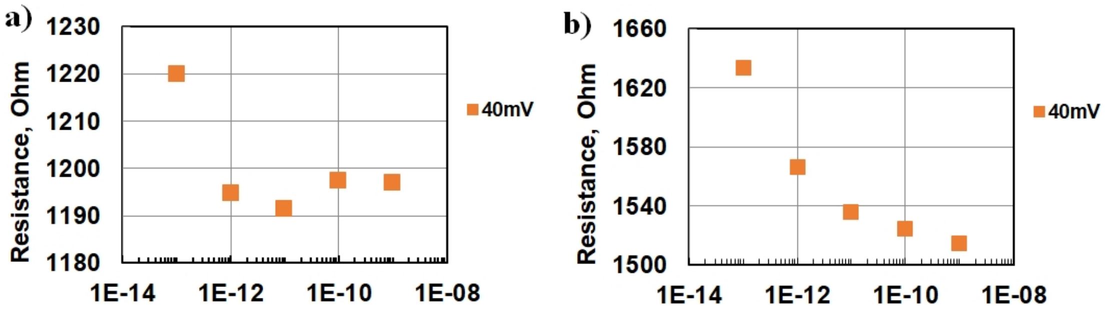

Figure S3. The resistance of the processed graphene chip versus the analyte (antigens of influenza B viruses) concentration in phosphate-buffered saline (PBS). When detecting viruses, DC voltage of 40 mV was applied to the graphene chip.
(a) Chip with LRRs on graphene.
(b) Chip without LRRs on graphene.
An improvement in sensitivity is observed for a graphene chip without LRRs, the resistance decreases by 120 Ohm (b), and for a processed graphene chip with LRRs (a) - only by 30 Ohm.
